# Unexpected Findings in Magnetic Resonance Enterography and Their Clinical Significance

**DOI:** 10.1155/2016/4020569

**Published:** 2016-03-29

**Authors:** Srivathsan Ravindran, Sarah Helen Hancox, Neil Barlow, Arthur Dunk, David Howlett

**Affiliations:** ^1^Digestive Disease Centre, Brighton and Sussex University Hospitals, Eastern Road, Brighton BN2 5BE, UK; ^2^Eastbourne District General Hospital, Eastbourne BN21 2UD, UK; ^3^Radiology Department, Eastbourne District General Hospital, Eastbourne BN21 2UD, UK; ^4^Gastroenterology Department, Eastbourne District General Hospital, Eastbourne BN21 2UD, UK

## Abstract

*Aims*. To identify the prevalence of colonic and extraenteric incidental findings in magnetic resonance enterography (MRE) and their clinical significance.* Methods*. We retrospectively analysed 470 MRE studies carried out between March 2012 and 2014. Incidental findings were defined as those not expected from or made apparent on the referral. MRE reports were reviewed for colonic and extraenteric findings, subcategorised into “clinically significant” and “insignificant.” Follow-up was identified from the electronic patient record.* Results*. The majority of MRE requests were made for inflammatory bowel disease (97%). In total, 114 incidental findings were noted in 94 (20%) scans performed. There were 29 “colonic” findings (25%) with 55% having a diagnosis of colitis. Out of 85 extraenteric findings, ovarian cysts (25%), renal cysts (10%), and abdominal lymphadenopathy (9%) were the commonest. Cumulatively, 59 cases were clinically significant (52%); of these, 30 findings were not previously diagnosed, amounting to 26% of all incidental findings. This led to intervention in seven patients.* Conclusions*. Incidental findings are common in MRE and there is a substantial proportion that is clinically significant and requires further investigation. There need to be stratification of risk and employment of local guidelines in order to achieve this.

## 1. Introduction

Over the past decade, small bowel imaging has developed from barium-based studies to detailed cross-sectional imaging. We are now at a point where inflammatory bowel disease (IBD) can be identified reliably and managed based on imaging. The recent European Crohn's and Colitis Organisation (ECCO) and European Society of Gastrointestinal and Abdominal Radiology (ESGAR) consensus guidelines highlight the importance of small bowel cross-sectional imaging as an adjunct to endoscopy in identifying intra- and extramural complications of IBD [1]. Its use has shifted from simple diagnosis to disease assessment, assessing therapeutic response and a noninvasive method of identifying occult disease in asymptomatic patients [2].

Small bowel magnetic resonance imaging (MRI) or MR enterography (MRE) is comparable in sensitivity and specificity to other small bowel imaging modalities, namely, computed tomography (CT), but without the risk of ionising radiation [1]. Recent trials, such as the METRIC study [3], are focussing on assessing the diagnostic accuracy of MRE and other modalities such as ultrasound, which avoid ionising radiation.

There is, however, an increasing subset of patients undergoing MRE that have unexpected extraintestinal pathology reported. Incidental findings have been extensively discussed in other forms of cross-sectional bowel imaging, particularly CT colonography. Reviews have identified a significant proportion of patients with extracolonic findings and a large percentage of these patients are further investigated as a result [4–6]. There is a general paucity in data regarding incidental findings in MRE. Within the past five years there have been a handful of patient series looking at this, all retrospective in nature [7–10]. Less emphasis has been placed on colonic findings in MRE; however their presence does play an impact on patient management and may preclude further investigation. In their patient population, Gee and Harisinghani [11] found MRE had a sensitivity of 88% for detecting colonic Crohn's disease when comparing MRE to colonoscopy.

The aims of this study were to identify the prevalence of colonic and extraenteric incidental findings in MRE, their clinical significance, and the subsequent effect of further investigation.

## 2. Materials and Methods

We retrospectively analysed findings from MRE studies that were conducted between March 2012 and March 2014 within East Sussex Healthcare NHS Trust (ESHT). All scans were performed at either Conquest Hospital or Eastbourne District General Hospital. Data was extracted from the picture archiving and communicating system (PACS) and there was a minimum period of six months of follow-up from the last scan. Studies were reported primarily by a Consultant Radiologist with specialist gastrointestinal imaging interest.

Individual reports for each study were obtained and reviewed for incidental findings by the primary author. Images were not rereviewed to avoid clinical discrepancies. Incidental findings were defined as findings not expected from or made apparent on the initial referral. Small bowel findings were excluded and the remainder were grouped into “colonic” or “extraenteric” findings. Extraenteric complications of IBD, for example, fistulae and abscesses, were not included within this analysis. Further subgroup analysis divided incidental findings into “significant” and “insignificant.” Findings that were significant were defined as those that would normally require further investigation or follow-up; this was agreed after repeated analysis by a Consultant Radiologist and Gastroenterologist, in keeping with local trust guidelines. Patient follow-up was conducted by reviewing a combination of the electronic patient record, lab data, pathology and histology reports, correspondence, and results of further imaging. Use of this data also allowed the analysis to include results and investigations that had been previously performed in patients to place incidental findings into context.

All MRE studies were performed using a 1.5T scanner. Patients are given 1.5 litres of mannitol solution to drink over 45 minutes beforehand, followed by intravenous Buscopan and gadolinium, assuming there are no contraindications. Routine scan imaging protocols were conducted as per current practice. A multiplanar combination of T1/T2 weighting and dynamic sequence and dynamic postcontrast methods were employed.

In total 470 MRE scans were performed between March 2012 and March 2014 at ESHT. Multiple scans in the same patient were excluded, taking the initial scan for analysis as follow-ups were less likely to comment on incidental findings that had already been reported. Additionally two scans were later excluded which had a finding of colitis on MRE as they also demonstrated small bowel Crohn's disease, thereby meaning the colonic finding would be expected. All other scans were included. 459 scans in total were analysed; the majority (*n* = 363, 79%) were to investigate suspected small bowel Crohn's disease. The evaluation of known Crohn's disease was an indication for 79 patients (17%). Other indications included small bowel obstruction, anaemia, and small bowel malignancy and, in total, these accounted for 3% of all referrals (*n* = 15).

## 3. Results

**Figure 1:**
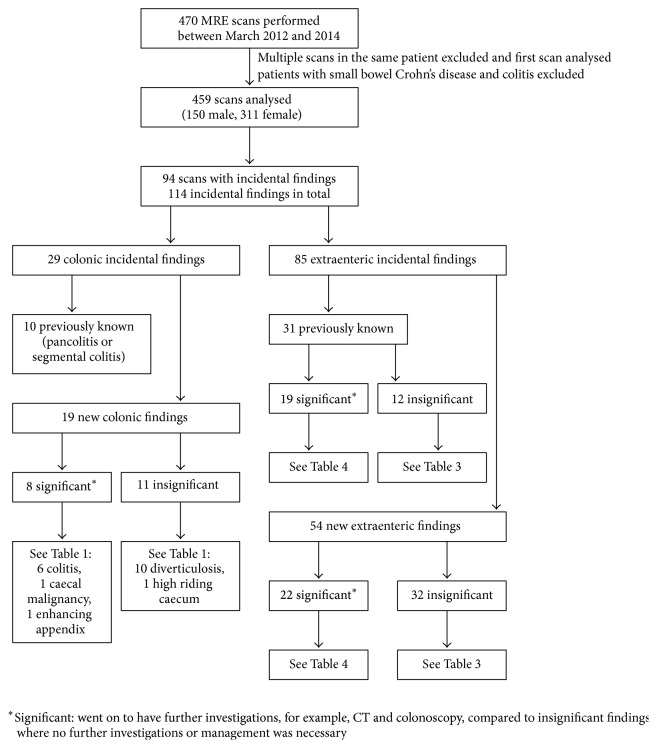
Flow diagram of patients identified for the study.

For all scans the mean age was 47.5 years (age range 7 to 83). There were 149 male (32%) and 310 female (68%) patients included in the study. We found that 94 scans (21%) had incidental findings. For these scans with incidental findings the mean age was also 47.5 years with mode and median of 48. From these studies 114 incidental findings were recorded: 29 were colonic (25%) and 85 were extraenteric (75%) (see Figure 1).

### 3.1. Colonic Findings

Of the 29 colonic incidental findings, there were 16 (55%) incidental findings of colitis. 10 (63%) of these were previously known having had positive diagnoses from colonoscopy and interestingly all were either pancolitis or segmental colitis. None of these cases had prior imaging and the colonoscopy results were not available to the radiologist at the time of reporting. For the other six (37%) patients with an incidental finding of colitis, this was a new diagnosis and all had a normal small bowel on imaging. Other colonic incidental findings comprised diverticulosis, caecal malignancy, and possible appendicitis (see Table 1).

**Figure 2:**
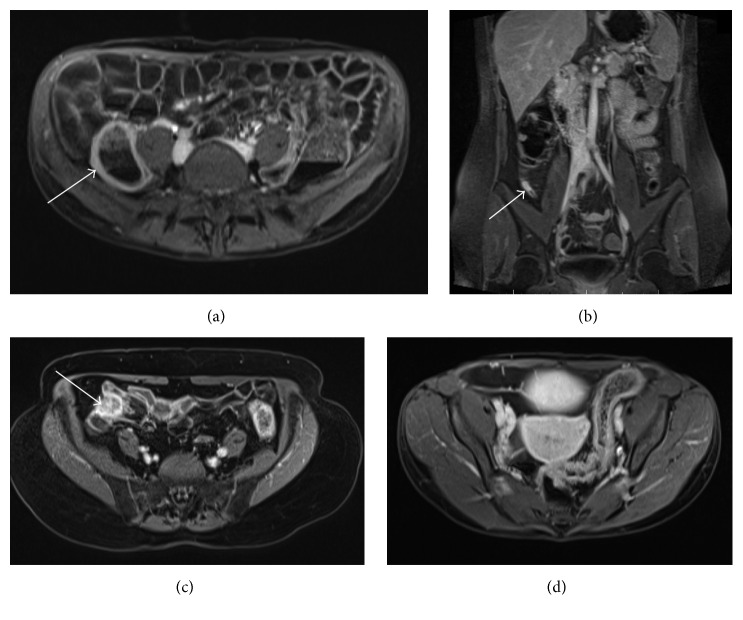
Colonic incidental findings: (a) enhancement of caecum (arrow) indicating colitis (axial T1 postgadolinium); (b) thickened, enhancing appendix (arrow, coronal T1 postgadolinium); (c) caecal cancer (arrow, axial T1 postgadolinium); (d) sigmoid colitis (axial T1 postgadolinium).

Of the findings that were not previously known about, eight were deemed significant (28%). Within this group, all patients went on to have further investigations. Six patients were found to have an incidental finding of colitis with previously normal investigations (colonoscopy or CT scan). Five out of these six patients with possible colitis were subsequently diagnosed with IBD on endoscopy with only one procedure revealing normal mucosa (see Figure 2). Furthermore one patient was further investigated for caecal malignancy by CT and colonoscopy, which resulted in a diagnosis of caecal carcinoma (see Figure 2). One other patient underwent CT and laparoscopy for an enhancing appendix tip, which was later excised.

### 3.2. Extraenteric Findings

**Figure 3:**
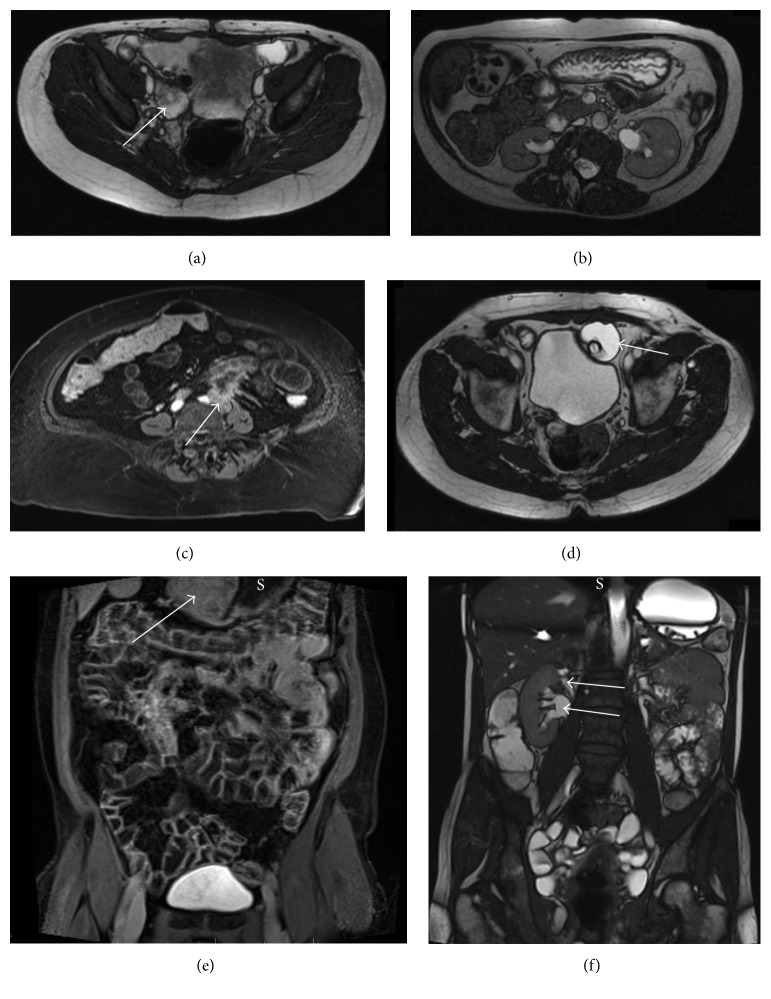
Extraenteric incidental findings: (a) right hydrosalpinx (arrow, axial T2); (b) gallstones in gallbladder (axial T2); (c) left ureteric tumour (arrow) with adherent jejunal loop (axial T1 postgadolinium); (d) suprapubic catheter balloon (arrow) lying outside bladder (axial T2); (e) gastric tumour (arrow; S: stomach; coronal T1 postgadolinium); (f) duplex kidney with upper and lower moieties identified (arrows, coronal T2).

There were 85 extraenteric findings, of which 31 were previously known about on previous imaging (36.5%, Table 2 and Figure 3). The overall commonest extraenteric findings were ovarian cysts (*n* = 21, 25%), renal cysts (*n* = 8, 9%), abdominal lymphadenopathy (*n* = 7, 8%), and gallstones (*n* = 7, 8%; see Table 2).

The majority of incidental findings were insignificant (*n* = 44, 52%; Table 3). Within this group the majority was gynaecological (ovarian cysts, fibroids; *n* = 15), renal (duplex systems, renal cysts; *n* = 12), and hepatobiliary (cholelithiasis, hepatic cyst; *n* = 6). There was one case of irregular inferior mesenteric artery vasculature which appeared to show a tortuous vessel; this was of no clinical consequence.

### 3.3. Significant Findings

From the 114 incidental findings found on MRE, in total there were 47 significant findings (41%) of which 30 were previously unknown (26% of total incidental findings). There were eight significant new colonic findings (27%; see Table 1) and 22 (73%; see Table 4) new extraenteric findings which all required further investigation/management. The eight significant new colonic findings were one caecal malignancy, one enhancing inflamed appendix, and six findings of colitis (either pancolitis or segmental colitis). The most common incidental new extraenteric findings were complex ovarian cysts, abdominal lymphadenopathy, and hydronephrosis (+/− nephrolithiasis).

In total five patients with extraenteric incidental findings underwent interventional procedures (4%). One underwent a gastrectomy for a gastric tumour which was confirmed as a gastrointestinal stromal tumour (GIST) by histology. Two patients underwent nephrolithotomies for renal stone disease. One patient was found to have pelviureteric junction (PUJ) obstruction and underwent JJ stenting and subsequent pyeloplasty. One patient was found to have a dermoid cyst, confirmed on transvaginal ultrasound, and underwent a laparoscopic cystectomy (see Table 4).

#### 3.3.1. Inappropriate Tests and False Positives

Four findings were deemed necessary for further investigation by the referring consultant despite not fitting “significant” criteria set by our study. These were all gynaecological in nature; three were adnexal cysts that were deemed physiological on MRE and one was a small uterine fibroid. One patient was found to have a possible sacral fracture but was subsequently investigated by X-ray and diagnosed with a sclerotic “bony island.”

Three cases were not further investigated despite having clinical significance. One was a 5 cm ovarian cyst that was not clearly benign. One was a patient with a dilated common bile duct to 12 mm with an obstructive calculus. Interestingly, this patient had no symptoms and normal liver functions tests; it was unclear whether the patient was offered further investigation. One case concerned a patient suspected to have a right iliac vein thrombus following MRE. No subsequent immediate investigation was performed but the patient presented five months later with breathlessness. A pulmonary embolism was subsequently diagnosed during this admission.

Other interventions included resiting a misplaced suprapubic catheter and liver biopsy for a patient with likely underlying cirrhosis and portal hypertension.

### 3.4. Cumulative Results

Out of a total of 114 incidental findings recorded, those that were unknown and significant amounted to 26% (*n* = 30; see Table 5).

## 4. Discussion

This study collected data from across two busy district general hospital sites. A relatively large cohort of patients underwent MRE within the two-year period. There was no reason apparent for the predominantly female population of the study (68%). Although the age range of patients having MRE was 7–83, young patients were outliers with a mean population age of 47.5 and there were no incidental findings in patients below the age of 18. The age range for patients with MRE incidental findings was 18–82. The mean age of patients with significant (colonic or extraenteric) findings was 57 years. This indicates that there are fewer incidental findings in younger patients and in paediatrics abdominal ultrasound scan could be used for evaluation. The major indication for imaging was the suspicion or evaluation of Crohn's disease. Similar studies have demonstrated this as well as a variety of other indications for MRE including assessment of coeliac disease, small bowel malignancy, protein-losing enteropathy, suspected appendicitis, and nonspecific abdominal pain [7–10].

In our series of 459 scans, we detected incidental findings (colonic and extraenteric) in 20% of patients. Similar incidence rates have been noted in the work by Jensen et al. [7] and Shrot et al. [9], who found 25% and 17% of patients had extraenteric incidental findings, respectively. However, this did not account for colonic findings, making comparison difficult. A larger series presented by Herfarth et al. [8] looked at 1154 MRE studies in 1006 patients over a 16-year period. Interestingly, a large proportion of patients, close to 60%, had extraenteric incidental findings (1113 findings in 600 patients, 59.6%). This may be explained by the authors' inclusion of extraenteric findings related to small bowel Crohn's disease, for example, abscesses, which are often discounted in other papers. Radhamma et al. [10] describe a much lower incidence rate of 3%. They explain that this may be a result of different reporting styles of radiologists; in their institution insignificant incidental findings such as renal or ovarian cysts are generally not reported.

Clearly the variety in indications for MRE, reporting of MRE, study design, series number, and patient population make comparing such studies difficult. There are however some similarities, particularly in the nature of incidental findings discovered. Ovarian cysts and gynaecological pathology are common with a frequency of 31% of extraenteric findings in our study. Others have had detection rates ranging from 15 to 25% of patients [7, 8]. Renal cysts and gallstones follow in order of decreasing frequency.

### 4.1. Significance of Findings

There has also been some attempt as to categorise the significance of findings. Within our series, there was no major difference between insignificant (52%) and significant (48%) extraenteric incidental findings. Within this latter group, 54% of findings were unknown, amounting to 25% of all extraenteric findings. Jensen et al. [7] identified that 72 (25%) of their 283 patients had extracolonic findings, of which 58 (20%) had previously unknown findings. Interestingly only five (2%) of these were classified as “potentially important.” Despite the marked, varied frequency of significant findings, this highlights a potential subset of patients that have significant pathology that would not otherwise be known about.

Interestingly, although the majority of unknown significant findings were investigated, three (14%) were not. The reasons behind this were unclear from documentation; however one could speculate that this may be due to lack of appreciation of their significance. Referring clinicians, usually Gastroenterologists or Gastrointestinal Surgeons, may not be fully aware of what nonenteric pathology warrants further investigation. This may also explain the inappropriate investigation of insignificant findings. A common link between these two groups is gynaecological pathology, particularly adnexal or ovarian cysts. Grand et al. [12] have produced a white paper on this very topic and provide guidelines for further management of incidental adnexal masses found on abdominal cross-sectional imaging. Local guidelines and department protocols may aid lines of investigation and the consideration of a multidisciplinary approach, such as that used in adrenal incidentalomas [13], may help.

### 4.2. MRE and Colitis

This study has demonstrated that 58% of colonic findings during MRE were related to large bowel inflammation and presumed colitis. Shrot et al. [9] confirmed reports of enhancing large bowel and likely colitis in 25% of 213 MRE studies performed. In our selection of patients, 10 of the 16 (63%) with a finding of colitis were previously known and diagnosed through colonoscopy. These patients were not excluded as there was no previous imaging or results of the colonoscopy available to the reporting radiologist in all cases. Additionally in all of these patients with an incidental finding of colitis, they had a normal small bowel on MRE. In the six patients that were not previously known to have colitis (normal previous imaging or colonoscopy) in our study, five were subsequently found to have disease on endoscopy following the finding of colitis on MRE.

These results point to the potential use of MRI in investigating colonic disease both alongside small bowel pathology and in isolation to assess the colon. Retrospective studies have shown over 84% sensitivity with the use of MRE in detecting colitis [11, 12, 14, 15]. Early studies investigating MRI colonography as a separate modality displayed poor sensitivity when compared to standard colonoscopy, particularly in mild colonic inflammation [16]. Other imaging modalities have shown similar data in terms of sensitivity of detection of colitis: CT 87%, abdominal ultrasound 75–91%, and capsule endoscopy 83% [15, 17, 18]. Colonoscopy remains the most specific investigation at 100%; however sensitivity is around 74% [18]. Cross-sectional imaging is gaining vast popularity in the assessment of IBD following diagnosis [11]. MRI is noninvasive and therefore does not convey the same risk as invasive endoscopy. This, coupled with less ionising radiation compared to CT imaging, makes MRI more appealing. Changes to MRI technology, contrast, enemas, and newer methods, including the “dark lumen” technique, have greatly improved the sensitivity and specificity of MRI colonography and are likely to progressively improve its diagnostic performance [1, 19].

### 4.3. Limitations

This study and many alike are limited by their retrospective nature. This is often confounded by the quality of the data expressed from reports, notes, and the electronic patient record. Reports by a variety of radiologists can alter the frequency of incidental findings through over- or underreporting. However, only a handful of reports in this series were reported by another radiologist. The minimum follow-up period of six months may also limit the interpretation and value of the study but every effort to include pending investigations and treatment has been made. An ideal study design would be prospective with a longer inclusion timeframe and follow-up.

### 4.4. Impact of Incidental Findings

We have demonstrated that incidental findings are common in MRE and that the majority of these are not significant. There remains however a proportion that is both new and significant and, in our study, this amounted to 26% of all incidental findings.

Clearly this will have some burden on clinicians as we use MRE more commonly in clinical practice. The most comparable literature regarding the clinical implications of incidental findings comes from CT colonography. Although it is useful in detecting early colonic malignancy, some authors have described this modality as being important in potentially detecting early abdominal malignancy [5]. Xiong et al. [20] looked into the financial cost of investigating extracolonic findings in routine CT colonography in symptomatic patients. Just over half of the patients examined (116 out of 225) had incidental findings; the cost of investigating and treating extracolonic lesions amounted to more than that for the original CT scan. There will always be some benefit to a small population in which incidental findings are discovered. Orme et al. [21] showed clear medical benefit in 1.1% of patients with incidental findings across a collection of imaging modalities. In our study, seven patients underwent clinical investigation and therapy which impacted their clinical outcome. Careful selection of patients who require further investigation is required and some form of stratification of potential risk should be employed to do this [4, 20].

## 5. Conclusions

The quantity of MRE access and burden will increase over the next decade. Its increasing use in disease reassessment and ability to reliably identify extracolonic complications of Crohn's disease and benefits of nonionising radiation in a young population make it a very useful tool in IBD.

There is a growing body of evidence showing that significant findings outside of the small bowel can be determined through MRE. Their prompt recognition can lead to altered patient outcomes and their potential impact should be a part of the discussion with patients prior to requesting MRE.

## Figures and Tables

**Table 1:** Colonic incidental findings.

Finding	Number	Significant?	Known finding?	Further investigation	Further result
Caecal malignancy	1	Y	0	CT, colonoscopy	Malignancy
Colitis (pan/segmental)	18	Y	12	Colonoscopy/sigmoidoscopy (*n* = 6)	Colitis (*n* = 5), normal mucosa (*n* = 1)
Diverticulosis	10	N	0	N/A	N/A
Enhancing appendix (?inflamed)	1	Y	0	CT, laparoscopy	Excision, fibrous tip of appendix
High riding caecum	1	N	0	N/A	N/A
Total	**31**		**12**		

**Table 2:** Extraenteric findings in order of frequency.

Finding	Number	Known
Ovarian cysts (complex)	11	4
Ovarian cysts (simple)	10	5
Renal cysts	8	0
Abdominal lymphadenopathy	7	4
Gallstones (simple)	6	3
Mesenteric fat hypertrophy	6	0
Hydronephrosis +/− nephrolithiasis	5	2
Uterine fibroids	5	1
Abdominal aortic aneurysm (>3 cm)	4	3
Duplex kidney	4	3
Gastric tumour	2	1
Hepatic cyst	2	0
Thrombosis	2	0
Abdominal wall hernia	1	0
Abnormal bone marrow	1	1
Avascular necrosis of femoral head	1	1
Epidural cyst	1	0
Free pelvic fluid	1	1
Gallstones (obstructed)	1	0
Hydrosalpinx	1	0
Irregular IMA vasculature	1	0
Misplaced suprapubic catheter	1	0
Portosystemic varices/irregular liver	1	0
Sacral fracture	1	0
Splenomegaly	1	1
Suspected ureteric tumour	1	1
Total	**85**	**31**
Percentage	**—**	**36.5%**

**Table 3:** Insignificant extraenteric findings.

Finding	Number	Known
Ovarian cysts (simple)	10	5
Renal cysts	8	0
Mesenteric fat hypertrophy	6	0
Gallstones (simple)	6	3
Uterine fibroids	5	1
Duplex kidney	4	3
Hepatic cyst	2	0
Abdominal wall hernia	1	0
Epidural cyst	1	0
Irregular IMA vasculature	1	0
Total	**44**	**12**
Percentage	**—**	**52.3%**

**Table 4:** Significant extraenteric findings, investigations, and results.

Finding	Number	Known	Investigation	Result
Ovarian cysts (complex)	11	4	TVUS (*n* = 6) No investigation (*n* = 1)	Physiological cyst (*n* = 3) Complex, haemorrhagic cyst (*n* = 1) Dermoid cyst and cystectomy (*n* = 1) Endometrial abnormality, awaiting laparoscopy (*n* = 1)

Abdominal lymphadenopathy	7	4	CT abdomen (*n* = 3)	Under surveillance with stable/normal limits (*n* = 3)

Abdominal aortic aneurysm	4	3	USS AA (*n* = 1)	AAA 40 cm (*n* = 1)

Hydronephrosis +/− nephrolithiasis	5	2	USS KUB (*n* = 2) CT KUB (*n* = 1) Functional scan (*n* = 3)	Nephrolithotomy (*n* = 2) JJ stent insertion & pyeloplasty (*n* = 1)

Gastric tumour	2	1	OGD (*n* = 1) Previous CT/OGD (*n* = 1)	Gastrectomy (*n* = 1) GIST (*n* = 1)

Thrombosis	2	0	CTPA (*n* = 1) CT abdomen (*n* = 1)	PE (*n* = 1) Iliac vein thrombus (*n* = 1)

Abnormal bone marrow	1	1	—	Known red cell aplasia

Avascular necrosis of femoral head	1	1	—	Known degenerative hip joint, awaiting replacement

Free pelvic fluid	1	1	Laparoscopy (*n* = 1)	Investigation declined by patient

Gallstone (obstructed)	1	0	No investigation	No intervention

Hydrosalpinx	1	0	TVUS pending^*∗*^	

Misplaced suprapubic catheter (SPC)	1	0	—	SPC replaced

Portosystemic varices/irregular liver	1	0	OGD & liver biopsy (*n* = 1)	Normal OGD (*n* = 1) Cirrhotic liver on biopsy (*n* = 1)

Sacral fracture	1	0	Pelvis XR (*n* = 1)	Sclerotic bony island, no fracture (*n* = 1)

Splenomegaly	1	1	—	—

Suspected ureteric tumour	1	1	—	Known ureteric malignancy

Total	**41**	**19**		

Percentage	**—**	**46.3%**		

Numbers in brackets denote frequency of investigation/diagnosis. AA: abdominal aorta; AAA: abdominal aortic aneurysm; CT: computed tomography; CTPA: CT pulmonary angiography; GIST: gastrointestinal stromal tumour; KUB: kidney, ureters, and bladder; OGD: oesophagogastroduodenoscopy; TVUS: transvaginal ultrasound scan; USS: ultrasound scan; XR: X-ray. ^*∗*^At time of analysis.

**Table 5:** Cumulative results.

Incidental findings	Significant	Insignificant	Total
Known	31 (27%)	12 (10%)	**43**
Unknown	30 (26%)	43 (37%)	**73**
Total	**61**	**55**	**116**

All percentages are of total 116.
